# Use of Lung Volume Recruitment Technique in Patients With Chronic Respiratory Disease Among Brazilian Health Professionals

**DOI:** 10.1155/pm/4073171

**Published:** 2025-01-10

**Authors:** Robert de Melo, Livia Alcantara, Max Sarmet, Nicole L. Sheers, David J. Berlowitz, Vinicius Maldaner

**Affiliations:** ^1^Post Graduation Department, Escola Superior de Ciências da Saúde (ESCS), Brasilia, Distrito Federal, Brazil; ^2^Human Movement and Rehabilitation Graduation Program, Unievangelica, Anapolis, Goias, Brazil; ^3^Department of Physiotherapy, Universidade de Brasilia, Brasilia, Brazil; ^4^Department of Physiotherapy, Secretaria de Saúde do Distrito Federal, Brasilia, Brazil; ^5^Department of Physiotherapy, Faculty of Medicine, Dentistry and Health Sciences, The University of Melbourne, Parkville, Victoria, Australia; ^6^Institute for Breathing and Sleep, Austin Health, Heidelberg, Victoria, Australia; ^7^Victorian Respiratory Support Service, Austin Health, Heidelberg, Victoria, Australia

**Keywords:** breath stacking, insufflation, lung inflation, lung volume recruitment, neuromuscular disease, respiratory muscles

## Abstract

**Background:** Lung volume recruitment (LVR) is a stacked-breath assisted inflation technique in which consecutive insufflations are delivered, without exhaling in between, until the maximum tolerable inflation capacity is reached. Although LVR is recommended in some neuromuscular disease guidelines, there is little information detailing when and how allied health professionals (AHPs) prescribe LVR.

**Objective:** This study is aimed at describing the use of LVR in practice across Brazil.

**Methods:** A cross-sectional e-survey (Sep–Nov 2023) explored LVR practices among qualified clinical or home care AHPs in Brazil. It gathered participant data on geographical region, profession, and experience. It delved into LVR specifics: clinical population and indications for use, prescription (frequency, dosage, and interfaces), related side effects, outcomes assessed, and combined therapies. Results were presented descriptively.

**Results:** One hundred two surveys (74 physical therapists (PTs) and 28 speech and language pathologists (SLPs)) from diverse locations were collected. LVR was predominantly prescribed for adults (57%), with the most common diagnosis being amyotrophic lateral sclerosis (84%). Changes in peak cough flow and vital capacity were the most common reasons for LVR prescription. Maximal insufflation capacity was reportedly measured by 58% of PTs and 22% of SLPs. Chest wall soreness and discomfort were the most common side effects, and many respondents did not provide warnings about potential side effects (42% PTs and 50% SLPs). The study highlighted common use of other respiratory therapy devices alongside LVR.

**Conclusion:** LVR is available in routine clinical and home care settings in Brazil. There is a lack of standardization regarding indications, prescription, and outcome measures among PTs and SLPs in Brazil. Clear recommendations and guidelines are needed to standardize these parameters, enabling more objective data and facilitating comparisons between centers.

**Summary**

• Lung volume recruitment (LVR) is commonly utilized in routine clinical and home care settings throughout Brazil, particularly by allied health professionals (AHPs), for patients with neuromuscular disease (NMDs). There are variations in prescription reasons and measurements made between physical therapists (PTs) and speech and language pathologists (SLPs).
• Clear recommendations and practical guidelines should better outline indications, prescription details, and side effects, enabling more objective data and facilitating comparisons between different centers.

## 1. Introduction

Respiratory muscle weakness, weak cough, and chest infections are a common feature of people living with NMDs and typically lead to respiratory impairment, and the associated clinical manifestations cause disability. It can cause dyspnea, mucus retention, atelectasis, and respiratory failure [1]. Recent guidelines recommend that a broad range of respiratory techniques be used in clinical practice, including the LVR technique. The LVR (*breath stacking*) technique utilizes a manual resuscitation bag with or without a one-way valve to increase the volume of air that can be inspired [2, 3].

LVR consists of consecutive bag compressions or insufflations without exhaling until the maximum tolerable inflation capacity is reached [4]. This incrementally augmented volume is defined as either the maximum or lung insufflation capacity (MIC or LIC), depending on the necessity (MIC) or not (LIC) for glottic closure. For the primary objective of lung expansion, assisted inflation is achieved followed by exhalation until functional residual capacity [5]. LVR enhances the expiratory airflow generated during cough, potentially facilitating the expulsion of mucus [4]. Furthermore, this technique has been advocated for the purpose of maintaining respiratory system and thoracic wall compliance, as well as decelerating the progression of respiratory system deterioration [3].

AHPs routinely incorporate LVR into their clinical practice when attending to patients with respiratory impairment. Despite consensus statements recommending LVR as adjunctive respiratory therapy [2, 3], it remains unclear how AHPs prescribe LVR and what AHP beliefs about outcomes, dosage, and clinical indications are. Thus, this study is aimed at describing the use of this technique in practice across Brazil. The specific objectives were to (i) gather information about current practice, patient population and reasons for prescribing LVR, type of equipment used, and recommended dosage and (ii) compare the differences in LVR practice between different AHP disciplines.

## 2. Methods

A cross-sectional electronic survey was open between 18th September and 3rd November 2023. Qualified AHPs working with chronic respiratory disease patients in clinical or home care settings in Brazil were invited to participate. Patients, caregivers, and relatives who performed LVR were excluded. An electronic link to the survey (REDCap) was disseminated through professional networks of the Brazilian Association of Respiratory Physical Therapy (ASSOBRAFIR, which has 830 members), the Respiratory Leaders' physical therapy and SLP groups (300 members), and via the networks' social media platforms (Facebook and Instagram). Participation was voluntary, and consent was obtained through online questionnaire completion. Ethical approval was granted by the Fundação de Ensino e Pesquisa em Ciências da Saúde (FEPECS) Reference Number 6.132.032.

The survey was developed by the study investigators (PTs) and SLPs with extensive working knowledge of LVR in chronic respiratory patients) and offered in Brazilian Portuguese (the English version is available in Supporting Information 1). Items included participant characteristics (profession, years of experience, and location) and LVR prescription practices (patient population, rationale for prescribing, recommended dosage prescribed, outcome assessment, and practical details). A repetition of LVR was defined as one complete assisted lung inflation [6].

### 2.1. Data Analysis

Returned surveys were reviewed to ensure that the respondents met the inclusion criteria. Only completed surveys were included in the analysis. Data were downloaded to Microsoft Excel and analyzed descriptively (percentages and means (standard deviations (SDs)) as appropriate).

## 3. Results

One hundred and two complete surveys were returned. The response rate could not be calculated owing to the distribution method and its unknown denominator.

**Figure 1:**
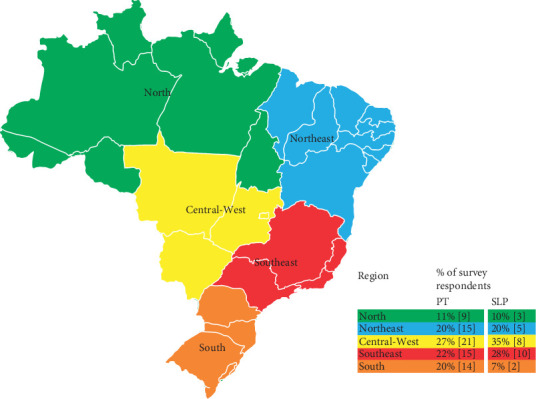
Geographic representation of survey respondents.

Respondents represented diverse geographical locations (Figure 1). Over half of the respondents were PTs (73%), followed by SLPs (27%). Experience of respondents varied between professions: < 2 years (32% of PTs and 46% of SLPs), 2–5 years (21% of PTs and 14% SLPs), 5–10 years (13% PTs and 36% SLPs), and > 10 years (34% of PTs and 4% of SLPs).

**Figure 2:**
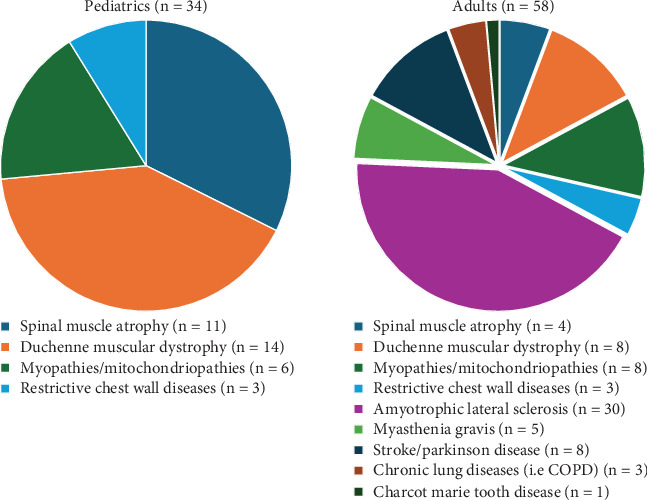
Subject diagnosis that uses LVR by the allied health professionals (ALHs).

Responders prescribed LVR in adults (57%), pediatrics (34%), or both populations (9%). LVR was prescribed primarily for people with NMDs, with all respondents reporting that they used LVR in this patient group, with amyotrophic lateral sclerosis (ALS) as the most common diagnosis. Some responders (11%) prescribed LVR for other diseases, such as stroke and Parkinson's disease (the survey allowed respondents to select more than one disease). See Figure 2 for full-subject diagnoses.

Table 1 illustrates reasons for prescribing LVR. Eighty-one percent of PTs used this technique for lung expansion and chest wall movement, whereas 78% of SLPs used LVR to improve voice. A peak cough flow (PCF) < 270 L/min was used by 92% of PTs and 79% of SLPs as a marker to initiate LVR. LVR via a face mask and one-way valve was used most by PTs (72% and 58%) whereas SLPs preferred the mouthpiece (79%) and no valve (86%). The majority of AHPs did not measure LIC or MIC to assess efficacy of LVR (42% PTs and 78% SLPs).

Table 2 describes the recommended prescription, type of interface and circuit, and patient education performed by each PTs/SLPs that answered our survey. The majority of AHPs recommended 10–15 repetitions of LVR per session, performed daily, with the number of compressions per maximal lung insufflation determined by patient comfort (Table 2). Forty-two percent of PTs and 50% of SLPs did not warn patients about side effects. Where side effects were discussed, chest wall soreness or discomfort was the most prevalent. LVR was most often prescribed in isolation, followed by combined with inspiratory muscle training (IMT) or mechanical insufflation–exsufflation (MI-E) devices.

## 4. Discussion

LVR has been investigated as an adjuvant treatment for patients with chronic respiratory disease, with its hypothesized benefits including maintaining the chest wall range of movement, improving cough effectiveness, and decreasing lung function decline [3, 7]. Its use in clinical practice is widespread and includes PTs and SLPs. This is the first survey of LVR use in clinical and home care settings in Brazil.

Access to respiratory therapy devices is challenging in Brazil, especially for its most vulnerable patients affected by a rare disease. Albuquerque et al. highlighted heterogeneity in access to PTs/SLPs according to the severity of the clinical condition, with motor physiotherapy being more frequent than respiratory physiotherapy for patients with spinal muscular atrophy (SMA). These dates were from a neuromuscular referral center located in Southern Brazil, one of the more developed regions of the country [8]. There is no national program in Brazil to deliver LVR kit/cough assistance devices and training professionals/caregivers to apply LVR in clinical practice. Additionally, the health care system's capacity to manage these patients with high needs and frequent respiratory infections will be increasingly strained [9]. In this context, health care must be developed to ensure better practices for chronic respiratory disease management.

The findings of this survey suggest that a considerable number of patients receiving LVR treatment are diagnosed with NMDs. The rationale for using LVR in NMD patients is based on the high occurrence of respiratory muscle weakness in this population, which is further compounded by their inability to routinely perform deep breathing [10]. A recent review by Sheers et al. found more than 30 cohort and randomized controlled trials (RCTs) on LVR in this population, most of which were cohort or prospective uncontrolled studies [4]. Only three RCTs of routine hyperinflation were performed, and they did not demonstrate improvement in lung function in NMD patients [11–13].

Our survey also referenced other populations, including those with restrictive chest wall disorders, such as cerebral palsy, stroke, chronic obstructive pulmonary disease (COPD), and Parkinson's disease. Simonds, Parker, and Branthwaite [14] examined the effects of intermittent hyperinflation in restrictive chest wall disease patients; they utilized noninvasive ventilation for mechanical insufflation instead of LVR. Unfortunately, no cohort studies or RCTs have been conducted to investigate the effects of LVR in these populations.

We found that PTs reported using the LVR technique in patients with COPD, despite this being contraindicated due to the prevalent hyperinflation in this population. A prior study on evidence-based practice (EBP) among PTs in Brazil revealed that many rely on expert opinion as a crucial factor in clinical decision-making, which conflicts with the use of EBP [15]. This same study also identified potential obstacles to EBP use among PTs, such as difficulties in obtaining full-text articles, language barriers in which articles are published, and challenges in interpreting results correctly. The e-survey conducted by our team revealed real-world results of clinical practice, and we believe that strategies should be developed to properly implement respiratory therapy techniques, focusing on individual patients, the correct execution of interventions, and methods to evaluate outcomes.

Like many other therapies, the reported LVR dosage is rarely standardized. In Brazil, LVR is recommended by neurologists from neuromuscular referral centers, but the detail regarding how to perform LVR (the “prescription”) is determined by the multidisciplinary team, especially PTs/SLPs. Differences among mode, interfaces, criteria for ending the insufflation, and dosage were found across our reported answers (Tables 1 and 2). Therefore, it is challenging to summarize the evidence about this technique of dose–response and its effectiveness in routine care. We propose that scientific societies should attempt to provide clearer guidance about settings for use of LVR into clinical practice.

We identified differences between PTs and SLPs regarding the prescription and utilization of LVR. Participants from both professions offered distinct rationales for prescription, employed diverse measures, administered varied dosages, and utilized different interfaces, as outlined in Table 2. Survey responses suggest that SLPs adopt LVR not only to improve respiratory outcomes but also to enhance voice and swallowing functions. Most SLPs (78%) reported using LVR to improve voice. Emerging evidence suggests a connection between phonation time and lung volumes across diverse populations, both of which are integral to voice function [16]. Numerous vocal therapy strategies employed by SLPs focus on enhancing lung volumes, consequently extending phonation time [17–19]. Therefore, this may be a contributing factor to the widespread adoption of LVR in clinical practice by SLPs. Similarly, PCF depends on generating subglottic expiratory air pressure, a crucial component of phonation and a significant upper airway metric regularly monitored in patients with NMD [5]. In summary, despite the current lack of substantial evidence supporting its efficacy, Brazilian SLPs may utilize LVR to enhance outcomes associated with voice function.

Research suggests that employing LVR techniques prior to swallowing could enhance respiratory reserves for swallowing in patients with ALS and other NMD [20, 21]. Furthermore, increased lung volume and subglottal pressure may contribute to increased expiratory airflow following swallow apnea [21, 22]. Based on the responses from SLPs, we propose conducting future studies to investigate the potential benefits of LVR in enhancing swallowing and voice outcomes across diverse populations.

Although the main reason for LVR prescription was the expansion of the lungs and movement of the chest wall, the main indication for this technique was PCF. This can be explained by the feasibility and affordability of the portable PCF assessment devices [23]. The slow and forced vital capacity (SVC and FVC) requires specialized testing equipment, trained clinicians, and travel to the referral neuromuscular center every 3 months, being an access barrier in the moderate/severe stages of the diseases, especially when travel is burdensome. The AHPs in Brazil often use the peak flow meter as the assessment device for cough effectiveness. Few professionals have access to portable spirometers and Wright ventilometer to evaluate lung volumes at home setting. To our knowledge, the best outcome to assess the effectiveness of LVR is not clearly defined, and further investigation is needed.

In our survey, we found a high number of AHPs that either did not use a one-way valve to perform LVR or did not assess LIC/MIC in their clinical practice. Unlike other countries (Australia, United States, and Canada), in Brazil, there are no kits with all the items (bag, mask, circuit, and one-way valve) available for purchase, and the one-way valve is not easily available [4]. Another probable factor is the lack of knowledge among AHPs regarding the differences between MIC and LIC and their importance in the longitudinal follow-up of these patients. Further studies should explore the importance of using these measures to prescribe and monitor patients in clinical practice.

Although uncommon, some studies have described side effects during the use of lung volume augmentation techniques. Katz et al. carried out an RCT on patients with Duchenne muscular dystrophy, and they have found reports of syncope and mild chest discomfort when using LVR [12]. Other side effects include fluctuations in blood pressure, bradyarrhythmia, and pneumothorax [24, 25]. AHPs that recommend LVR for their patients should advise them about the potential benefits and harms of routine LVR use.

Other therapies have been applied in combination with LVR in clinical practice in Brazil (Table 2). The most common adjunctive therapies described were MI-E and respiratory muscle training, including inspiratory/expiratory muscle training (IMT/EMT). The growing body of evidence on the use of EMT to treat dysphagia in neurodegenerative diseases is evident, and as many SLPs use the technique and given that one of the main reasons for its prescription was dysphagia, the addition of EMT may be justified in clinical practice [26–28]. The need for further investigation into the use of IMT/EMT in LVR routine care is warranted.

The MI-E devices are an available method for performing assisted inflations in routine care, [29]; however, they are expensive, heavy, and not very portable [30]. While certain MI-E devices may be accessible or funded in certain jurisdictions, their cost remains a significant obstacle in many other settings. Moreover, they often necessitate specialized expertise to install and configure, as well as ongoing technical assistance and maintenance, which may present additional barriers to access [4]. We did not investigate the reasons for use of MI-E in conjunction with LVR, but we hypothesized that AHPs use LVR with the aim being lung expansion and chest wall movement and the MI-E as a cough augmentation technique to clear the lungs. On the other hand, the LVR is a portable technique used as a cough augmentation technique when out in the community, compared to MI-E in home [31]. Therefore, patients can use the techniques as complementary forms, with one being used in the home environment and the other allowing greater patient mobility, as well as MI-E availability limitations, due to electrical problems and/or the MI-E device.

There are limitations to this survey that need to be acknowledged. Due to the distribution method, it was not possible to calculate a response rate for the survey. As with any self-report survey, the responses may not reflect all practices. Differences in local health care practice and funding of devices such as MI-E may have influenced prescription and use patterns, but we were unable to assess the impact of these potential economic and other factors on the questionnaire responses. Despite these limitations, this survey described current practices with LVR within a wide geographical representation of Brazil.

## 5. Conclusion

LVR is widely available in routine clinical and home care settings across Brazil. The AHPs use it in patients with NMD preferably, and PTs and SLPs showed differences in reasons and outcome measures for prescription. There is a lack of standardization regarding measures, indications, and dosage among PTs in Brazil. Recommendations and practical guidelines should make clear the indications and prescription details for regular use of the technique, so that we can have more objective data and compare results between different centers.

## Figures and Tables

**Table 1:** Characteristics of LVR prescription.

**Reasons for LVR prescription**	**Physical therapist (** **n** = 74**)**	**Speech and language pathologists (** **n** = 28**)**
To expand the lungs and move the chest wall (“range of movement” exercise)—*n* (%)	60 (81%)	19 (68%)
Neuromuscular disease confirmed diagnosis	45 (61%)	14 (50%)
To improve cough effectiveness (proximal airway clearance)—*n* (%)	30 (40%)	11 (39%)
To clear secretions from the lungs (peripheral airway clearance)—*n* (%)	12 (16%)	12 (43%)
To prevent chest infections—*n* (%)	11 (15%)	5 (18%)
Improve voice—*n* (%)	10 (13%)	22 (78%)
Others (dysphagia)	5 (7%)	3 (12%)
*Measures adopted to prescribe LVR by AHPs*		
PCF < 270 L/min	68 (92%)	22 (79%)
PCF < 160 L/min	40 (54%)	18 (64%)
FVC and/or SVC—*n* (%)	42 (57%)	9 (32%)
Symptoms of respiratory failure—*n* (%)	38 (41%)	8 (28%)
MIP/SNIP—*n* (%)	30 (40%)	11 (39%)
Others (dysphagia scales, PSG)	5 (7%)	12 (43%)
*Measurement of LIC/MIC*		
Yes	43 (58%)	6 (22%)
No	31 (41%)	22 (78%)
*Frequency of LIC/MIC*		
Monthly—*n* (%)	3 (4%)	—
Weekly—*n* (%)	4 (5%)	—
Every clinical visit—*n* (%)	24 (32%)	4 (14%)
3 months—*n* (%)	12 (16%)	2 (8%)
Did not measure—*n* (%)	31 (41%)	22 (78%)

Abbreviations: AHP, allied health professionals; FVC, forced vital capacity; LIC, lung insufflation capacity; LVR, lung volume recruitment; MIC, maximal insufflation capacity *n*, number of responses; MIP, maximal inspiratory pressure; PCF, peak cough flow; SNIP, sniff nasal inspiratory pressure; SVC, slow vital capacity.

**Table 2:** Guidance for patients to perform LVR.

**Mode**	**Physical therapist (** **n** = 74**)**	**Speech and language pathologists (** **n** = 28**)**
Daily—*n* (%)	53 (72%)	18 (64%)
2× per week—*n* (%)	4 (5%)	2 (7%)
3× per week—*n* (%)	3 (4%)	2 (7%)
5× per week—*n* (%)	9 (12%)	4 (14%)
Any time/as often as they decide—*n* (%)	3 (3%)	2 (4%)
Depends on the reason for prescribing	2 (2%)	2 (4%)
*Best criteria for end the insufflation*		
1–3 bag compressions—*n* (%)	2 (3%)	1 (3%)
3–5 bag compressions—*n* (%)	4 (5%)	1 (3%)
Patient's tolerance	43 (58%)	15 (53%)
Maximum expansion of the chest cavity (visual analysis)	9 (12%)	3 (14%)
Until reaching a predetermined pressure limit (monitored by the manometer)—*n* (%)	16 (21%)	8 (28%)
*Repetitions of LVR*		
1–5 reps—*n* (%)	3 (2%)	—
5–10 reps—*n* (%)	4 (5%)	1 (3%)
10–15 reps—*n* (%)	33 (45%)	15 (53%)
15–20 reps—*n* (%)	18 (27%)	4 (14%)
Any reps—*n* (%)	16 (21%)	8 (28%)
*Preferred LVR interface*		
Mouthpiece	33 (45%)	22 (79%)
Mask	53 (72%)	18 (64%)
Endotracheal tube/tracheal cannula	33 (45%)	2 (7%)
*One-way valve*		
Yes	43 (58%)	4 (14%)
No	31 (42%)	24 (86%)
Depends on the reason for prescribing	2 (2%)	2 (4%)
*Respiratory therapy devices associated*		
Mechanical insufflation–exsufflation—*n* (%)	30 (26%)	7 (25%)
Flutter/acapella—*n* (%)	12 (16%)	4 (14%)
Inspiratory muscle training—*n* (%)	20 (27%)	2 (8%)
Expiratory muscle training—*n* (%)	10 (13%)	1 (4%)
Incentive spirometry—*n* (%)	8 (10%)	3 (10%)
Did not use any device—*n* (%)	31 (42%)	14 (50%)

Abbreviations: LVR = lung volume recruitment, *n* = number of responses from 102 participants/two main professional discipline groups.

## Data Availability

Data sharing is not applicable to this article as no datasets were generated or analysed during the current study.
