# Supplementary material for: Use of Lung Volume Recruitment Technique in Patients With Chronic Respiratory Disease Among Brazilian Health Professionals
Source: Pulm Med. 2025 Jan 10;2025:4073171. doi: 10.1155/pm/4073171 (PMC11748743; doi:10.1155/pm/4073171)
Supplement: Supporting Information — Additional supporting information can be found online in the Supporting Information section. English version of the survey applied for the allied health professionals. [file 4073171.f1.pdf]

# Lung Volume Recruitment (LVR) Technique

**WHY IS THIS STUDY BEING DONE?**

This survey aims to register the current practice of monitoring of the patient initiated on lung volume recruitment in centers that have both clinical and academic experience in respiratory care of this neuromuscular patient population. We welcome responses from all professionals. The purpose of the survey is to gather information around current practice i.e., which countries, side effects, reasons for prescription and dosage. This will help to identify to explore current practice.

**WHAT IS REQUIRED DURING THIS STUDY?**

If you choose to take part in the project you will be asked to complete an online questionnaire to rate the importance of LVR which help neuromuscular disease patients with respiratory care.

You will be asked for your opinion of 17 questions related to objective and subjective criteria for indicating therapy.This list was generated from a review of existing studies and interviews with patients with neuromuscular disease.

The data collected in this survey will be anonymized and analyzed by a group of specialists. We aim to publish the results by the end of 2023.

The survey will take around 10 minutes to complete. We thank you for you participation in this survey.

Vinicius Maldaner PT, PhD, Prof. David Berlowitz and Nicole Sheers PT, PhD

* Indica uma pergunta obrigatória

1. E-mail *

_______________________

2. Which country / where do you practice?

*Marcar apenas uma oval.*

- ( ) Brazil
- ( ) Australia

3. Job title

*Marcar apenas uma oval.*

- Physical Therapist / Physiotherapist
- Nurse
- Physician/Doctor
- Speech Therapist
- Occupational Therapist
- Outro: ____________________

4. How many years of experience do you have with chronic respiratory patients?

*Marcar apenas uma oval.*

- less than 2 years
- 2-5 years
- 5-10 years
- higher than 10 years

5. Do you prescribe lung volume recruitment (LVR) for all people you see with a neuromuscular disease (NMD)?

*Marcar apenas uma oval.*

- Yes
- No

6. For what reason do you prescribe LVR?

*Marque todas que se aplicam.*

- [ ] To improve cough effectiveness (proximal airway clearance)
- [ ] To clear secretions from the lungs (peripheral airway clearance)
- [ ] To expand the lungs and move the chest wall ("range of movement" exercise)
- [ ] To prevent chest infections
- [ ] To practice the technique, so that it can be used during a chest infection
- [ ] Outro: ______________________

7. What measures do you use for LVR prescription? Please select all that apply:

*Marque todas que se aplicam.*

- [ ] Forced vital capacity (FVC) < 80% predicted/Slow vital capacity < 80% predicted
- [ ] Forced vital capacity (FVC) < 50% predicted / Slow vital capacity < 50% predicted
- [ ] MIP/SNIP < 40 cmH2O
- [ ] confirmed NMD diagnosis
- [ ] Peak Cough Flow (PCF) < 270 l/min
- [ ] Peak Cough Flow (PCF) < 160 l/min
- [ ] presence of symptoms (for example: orthopnea, breathlessness, difficulty clearing sputum)
- [ ] history of chest infection/s
- [ ] Other (please describe):
- [ ] Outro: ______________________

8. What patient groups or diagnoses do you prescribe LVR for?

*Marque todas que se aplicam.*

- [ ] Amyotrophic Lateral Sclerosis (ALS) / Motor Neuron Disease (MND)
- [ ] Muscular Dystrophy
- [ ] Spinal Muscular Atrophy
- [ ] Myasthenia Gravis
- [ ] Lung diseases (i.e COPD, bronchiectasis)
- [ ] Myopathies
- [ ] Mitochondriopathies
- [ ] Restrictive chest wall disease (for example, kyphoscoliosis)
- [ ] Stroke
- [ ] Parkinson disease
- [ ] Outro: ____________________

9. How often do you recommend your patient performs LVR?

*Marcar apenas uma oval.*

- ( ) daily
- ( ) 2 times per week
- ( ) 3 times per week
- ( ) 5 times per week
- ( ) any time / as often as they decide
- ( ) more than once a day
- ( ) depends on the reason for prescribing
- ( ) Outro: ____________________

10. What criteria do you use to end the resultant lung inflation?

*Marque todas que se aplicam.*

- [ ] 1-3 stacking breaths (1-3 bag squeezes or compressions)
- [ ] 3-5 stacking breaths (3-5 bag squeezes or compressions)
- [ ] patient tolerance
- [ ] full chest wall expansion (based on observation)
- [ ] pre-set pressure limit reached (pressure release valve or manometer in circuit)
- [ ] Outro: ______________________

11. How many sets of LVR described above do you prescribe for your patient for each session?

*Marcar apenas uma oval.*

- ◯ 5-10 sets
- ◯ 10-15 sets
- ◯ 15-20 sets
- ◯ any sets

12. What interface do you use to perform LVR?

*Marcar apenas uma oval.*

- ◯ mouthpiece
- ◯ oronasal mask
- ◯ endotracheal tube/tracheal cannula

13. Do you use a one-way directional valve in the LVR circuit?

*Marcar apenas uma oval.*

- ◯ Yes
- ◯ No

14. How often do you measure the maximal LVR-assisted insufflation capacity (lung insufflation capacity, LIC or maximal insufflation capacity, MIC)?

*Marcar apenas uma oval.*

- monthly
- weekly
- every 3 months
- at every clinical visit
- I do not measure it
- Other (please describe):

15. How else do you assess the effectiveness of LVR over time?

_______________________________________________
_______________________________________________
_______________________________________________
_______________________________________________
_______________________________________________

16. Who performs the LVR technique when at home? *

*Marque todas que se aplicam.*

- [ ] the patient (independently)
- [ ] paid caregivers
- [ ] parents/relatives
- [ ] health professionals
- [ ] Outro: _______________

17. What side effects do you educate your patient about? Select all that apply *

*Marque todas que se aplicam.*

- [ ] Lightheadedness
- [ ] Dizziness
- [ ] Chest wall soreness or discomfort
- [ ] Bloating / swallowing air / aerophagia
- [ ] Pneumothorax
- [ ] Outro: ______________________

18. What does respiratory therapy device use along LVR? *

*Marque todas que se aplicam.*

- [ ] Flutter, Acapella
- [ ] PEP and bubble PEP
- [ ] inspiratory load device (Power Breathe, Threshold IMT)
- [ ] Incentive Spirometry (Voldyne, Respiron)
- [ ] Outro: ______________________

Este conteúdo não foi criado nem aprovado pelo Google.

Google Formulários
